# Trends of Overweight and Obesity in Male Adolescents: Prevalence, Socioeconomic Status, and Impact on Cardiovascular Risk in a Central European Country

**DOI:** 10.1007/s11695-021-05867-z

**Published:** 2022-01-18

**Authors:** Lisa Gensthaler, Daniel M. Felsenreich, Julia Jedamzik, Jakob Eichelter, Larissa Nixdorf, Christoph Bichler, Michael Krebs, Bianca Itariu, Felix B. Langer, Gerhard Prager

**Affiliations:** 1 Division of Visceral Surgery, Department of General Surgery, Medical University of Vienna, Waehringer Guertel 18-20, 1090 Vienna, Austria; 2 Division of Endocrinology, Department of Internal Medicine, Medical University of Vienna, 1090 Vienna, Austria

**Keywords:** Adolescents, Overweight, Obesity, Socioeconomic status, Cardiovascular risk, Bariatric surgery

## Abstract

**Background:**

Obesity is one of the most important health-related problems of the twenty-first century. Data on its prevalence in Austria remain scarce. Aim of this study was to determine current trends of overweight and obesity, associated comorbidities and socioeconomic status in all 18-year-old male Austrian citizens, and its potential impact on the demand for bariatric surgery in the future.

**Methods:**

Data from compulsory military conscription examinations in all 18-year-old males from 2003 to 2018 were obtained from the Federal ministry of Defense’s database. Measurements of height, weight, and subsequent body mass index (BMI) calculations in 874, 220 adolescents were subdivided into yearly cohorts. Comorbidities, educational status, and nicotine abuse were evaluated.

**Results:**

Mean BMI increased from 22.0 ± 3.95 kg/m^2^ in 2003 to 22.8 ± 4.69 kg/m^2^ in 2018 (*p* < 0.001). Overweight and obesity °I–III increased from 15.3%, 4.2%, 1.2%, and 0.4% (2003) to 20.4%, 7.1%, 2.5%, and 0.8% (2018), respectively. Cardiovascular risk, reflected by the waist-to-height ratio, increased significantly over time (*p* < 0.0001). Additionally, data showed a significant association of nicotine abuse in overweight or obese adolescents (*p* < 0.0001). Significantly less adolescents with obesity graduated from high school (*p* < 0.0001). Overall, 25.7% of adolescents with obesity were considered ineligible for military service.

**Conclusions:**

BMI and cardiovascular risk steadily increased over the last 15 years in Austrian male adolescents. A significant shift from normal weight to overweight was observed, while higher obesity classes doubled over this observational period. This study also revealed a significant association of BMI and lower educational status.

**Graphical Abstract:**

Trends of overweight and obesity in male adolescents: prevalence, socio-economic status and impact on cardiovascular risk in a central European country 
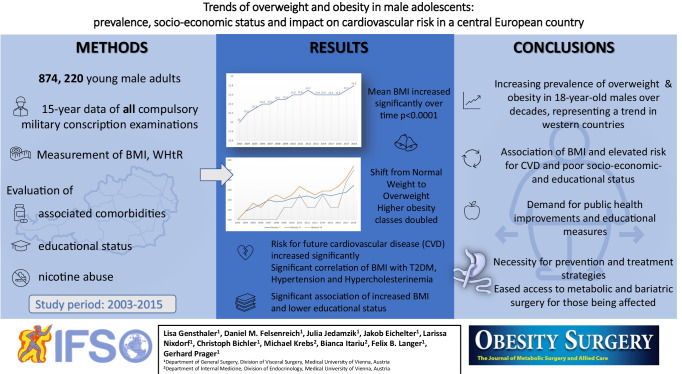

## Introduction

Obesity and its associated comorbidities represent one of the factors most compromising for human health in the twenty-first century. While predictions by the National Health and Nutrition Examination Survey (NHANES) [1] estimate that obesity has reached a plateau, the World Health Organization (WHO) still reports a significant increase of obesity in both men and women, with rates almost tripled within the last 30 years [2]. In 2016, 1.9 billion adolescents with overweight (39%) (body mass index (BMI) > 25 kg/m^2^) were identified worldwide. Of those, 650 million (13%) young adults presented with BMI > 30 kg/m^2^. Obesity also affects children: 38 million children under 5 years and 340 million children between 5 and 18 years of age were reported to be either overweight or obese [3, 4].

Obesity is associated with several comorbidities, such as type 2 diabetes mellitus (DM II), cardiovascular disease (CVD), sleep apnea, and musculoskeletal disorders. Furthermore, the risk of developing hormone-active cancers is associated with and triggered by obesity [5–7]. Especially in younger patients, the outcome of obesity-associated cardiovascular or cerebrovascular incidents is inferior compared to older patients [7]. These comorbidities remain the number one cause of death in Western countries and represent a logistical and financial burden for the health care system [8, 9]. Disadvantages of existing databases reporting the worldwide or regional development of overweight and obesity are that they are based on estimations and incomplete data, often focusing on small geographic regions, population subgroups [10, 11], or self-reported data [12].

The objective of this study was to evaluate current trends in weight, BMI, and comorbidities in a Western European country with a comprehensive database of complete and centrally reported data. Weight, BMI, and comorbidities of all 18-year-old male Austrians between 2003 and 2018 (compulsory military conscription examinations) were grouped and correlated with socioeconomic variables.

## Material and Methods

Data from the compulsory military conscription examinations in all 18-year-old male Austrians between 2003 and 2018 were obtained. Data acquisition was performed in cooperation with the Federal Ministry of Defense. The prospectively collected data of 874,220 male adolescents that were examined within the observational period, was evaluated at the Medical University of Vienna retrospectively. This number represents > 95% of Austrian male adolescents. Only those suffering from severe mental or physical disorders, thus being unable to participate, were excluded from the obligatory examinations. Data collection and blood sampling were highly standardized by well-instructed military service members. A sub-analysis, focusing on collected data between 2006 and 2010, was previously published by Poglitsch et al. [13].

### Weight and BMI

Data included were height, weight, BMI, conscripts’ health status, medical history, and clinical status. The subjects were allocated to the following subgroups defined by the WHO guidelines based on the BMI: underweight (BMI ≤ 18.50 kg/m^2^), normal weight (BMI 18.50– < 24.99 kg/m^2^), overweight (BMI 25.00–29.99 kg/m^2^), and obesity (obesity °I: BMI 30.00–34.99 kg/m^2^, obesity °II: BMI 35.00–39.99 kg/m^2^, and obesity °III: BMI ≥ 40.00 kg/m^2^) [14].

### Comorbidities

Data on comorbidities, encoded with the International Statistical Classification of Diseases and Related Health Problems (ICD) 9 and 10 as well as laboratory and vital parameters, were collected as well. To identify any risk for CVD, the waist-to-height ratio (WHtR), defined as the proportion of abdominal girth to height, was used with a cut-off of 0.5 [4]. Hyperglycemia, identified by an elevated fasting blood sugar limit (> 125 mg/dl), hypercholesterinemia (> 200 mg/dl), and increased systolic blood pressure (> 140 mmHg) were correlated with BMI. Further parameters to identify those at risk for developing a metabolic syndrome, such as hypertriglyceridemia (> 150 mg/dl), low HDL cholesterol (< 40 mg/dl), and abnormal abdominal girth (> 94 cm), were evaluated as well.

### Education and Socioeconomic Status

Nicotine consumption and educational status, as appropriate indicators for socioeconomic status, were evaluated and subsequently correlated with the BMI of these young male adults. The educational status was subdivided into groups, depending on the young male adult’s educational certificate and status of graduation (1a, no compulsory education; 1b, compulsory education but no high school graduation; 2, high school graduation or higher). The correlation of the educational status (1a, 1b, 2) and BMI was analyzed using furthermost numbers by one-way ANOVA statistical analysis.

The Ethics Committee, representing the local Institutional Review Board (IRB), approved this study (approval number: 2261/2019). Since the participants’ records were anonymized prior to analysis and due to the retrospective study design, no informed consent was required.

### Statistical Analysis

Descriptive data are shown as mean values with standard deviation (SD), as percentage, if appropriate, or as 95% confidence intervals (CI). To determine group differences of data for normally distributed variables, t-tests and chi-square tests were performed. Categorical data were described with absolute values and relative frequencies. The main aim of the study was to analyze changes of mean BMI over time, which was modeled by linear cox-regression models and ANOVA test. *P*-values of < 0.05 were considered statistically significant when comparing values in univariate analysis. Statistical analyses were performed using SPSS 25.0 for Mac (IBM Inc., Armonk, NY); figures were created with Microsoft Excel.

## Results

Data from all 18-year-old male Austrians eligible for military conscription examinations from 2003 to 2018 were obtained from the Federal Ministry of Defense database and included in this retrospective study. Ranging from 44,911 in 2003 to a maximum of 58,774 in 2010, 874,220 subjects were included in total, representing almost all Austrian male adults at the age of 18.

### Weight and BMI

**Fig. 1:**
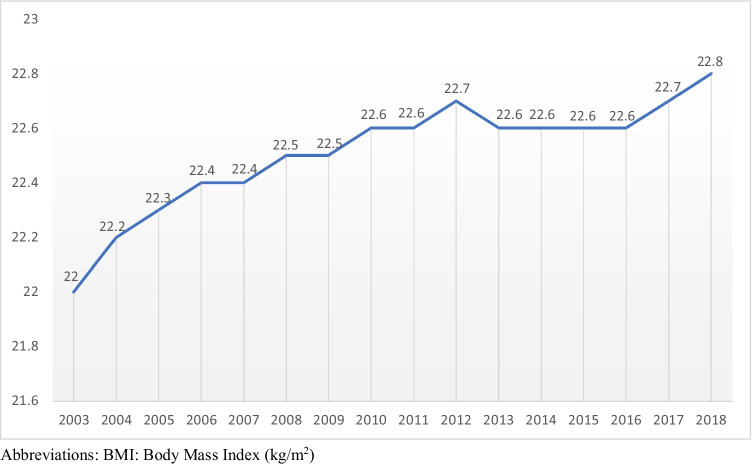
Mean BMI - Distribution 2003-2018

**Fig. 2:**
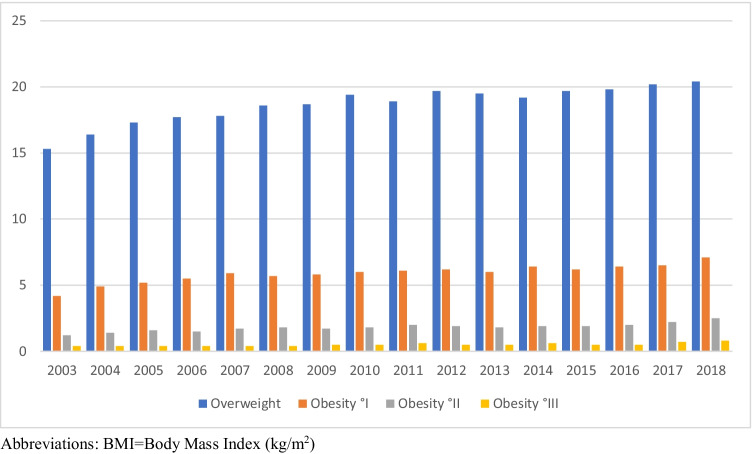
Distribution of overweight and obesity °I-III in 18-year-old males, 2003-2018; n=237,730 (27.2%)

**Fig. 3:**
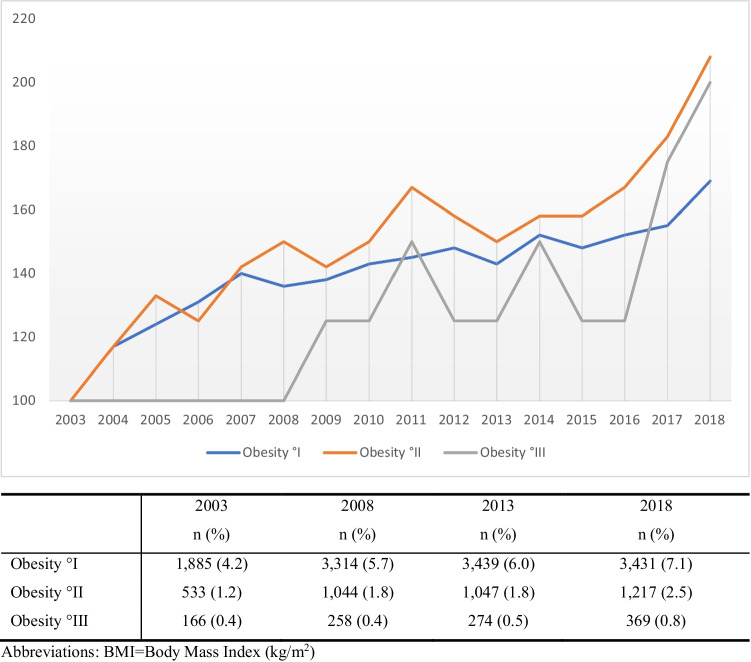
Prevalence of obesity °I-III in Austria in 18-year-old males 2003-2018; n= 71,856 (8.2%)

The mean BMI increased from 22.0 ± 3.95 kg/m^2^ in 2003 to 22.8 ± 4.69 kg/m^2^ in 2018 (*p* < 0.001) (Fig. 1). Linear regression models highlighted an average increase in BMI of 0.053 kg/m^2^ per year (*p* < 0.0001). In general, overweight and obesity °I–III increased from 15.3%, 4.2%, 1.2%, and 0.4% in 2003 to 20.4%, 7.1%, 2.5%, and 0.8% in 2018, respectively, as shown in Table 1 and Fig. 2. In fact, a shift from normal weight to overweight may be observed over the past 15 years. Figure 3 shows the distribution of obesity °I–III in detail (Table 3).

**Table 1:** BMI distribution in Austria in all 18-year-old males 2003–2018, *n* = 874,220(%)

Year	Number (n)	Underweight BMI < 20 *n* (%)	Normal weight BMI ≥ 20–25 *n* (%)	Overweight BMI ≥ 25–30 *n* (%)	Obesity °I BMI ≥ 30–35 *n* (%)	Obesity °II BMI ≥ 35–40 *n* (%)	Obesity °III BMI ≥ 40 *n* (%)
2003	44,911	9,738 (21.7)	25,702 (57.2)	6,887 (15.3)	1,885 (4.2)	533 (1.2)	166 (0.4)
2004	49,721	10,224 (20.6)	28,054 (56.4)	8,138 (16.4)	2,413 (4.9)	717 (1.4)	175 (0.4)
2005	53,122	10,381 (19.5)	29,772 (56.0)	9,168 (17.3)	2,751 (5.2)	848 (1.6)	202 (0.4)
2006	57,765	10,902 (18.9)	32,369 (56.0)	10,199 (17.7)	3,150 (5.5)	891 (1.5)	254 (0.4)
2007	58,649	10,887 (18.6)	32,606 (55.6)	10,432 (17.8)	3,467 (5.9)	1,013 (1.7)	244 (0.4)
2008	57,750	10,294 (17.8)	32,090 (55.6)	10,750 (18.6)	3,314 (5.7)	1,044 (1.8)	258 (0.4)
2009	58,663	10,479 (17.9)	32,531 (55.5)	10,968 (18.7)	3,384 (5.8)	1,010 (1.7)	291 (0.5)
2010	58,774	10,223 (17.4)	32,293 (54.9)	11,392 (19.4)	3,510 (6.0)	1,061 (1.8)	295 (0.5)
2011	58,212	10,327 (17.7)	31,819 (54.7)	11,018 (18.9)	3,551 (6.1)	1,152 (2.0)	345 (0.6)
2012	57,640	10,268 (17.8)	30,982 (53.8)	11,368 (19.7)	3,592 (6.2)	1,117 (1.9)	313 (0.5)
2013	56,921	10,379 (18.2)	30,683 (53.9)	11,099 (19.5)	3,439 (6.0)	1,047 (1.8)	274 (0.5)
2014	56,721	10,385 (18.3)	30,363 (53.5)	10,912 (19.2)	3,642 (6.4)	1,106 (1.9)	340 (0.6)
2015	53,282	10,133 (19.0)	28,041 (52.6)	10,492 (19.7)	3,305 (6.2)	1,036 (1.9)	275 (0.5)
2016	51,237	9,797 (19.1)	26,779 (52.3)	10,137 (19.8)	3,266 (6.4)	1,022 (2.0)	236 (0.5)
2017	52,211	9,837 (18.8)	26,910 (51.5)	10,559 (20.2)	3,402 (6.5)	1,155 (2.2)	348 (0.7)
2018	48,641	9,179 (18.9)	24,537 (50.4)	9,908 (20.4)	3,431 (7.1)	1,217 (2.5)	369 (0.8)

Abbreviations: *BMI* body mass index (kg/m^2^)

### Comorbidities

A total of 237,730 out of 874,220 (27.2%) were identified as overweight or obese °I–III individuals and analyzed regarding a potential association of overweight and obesity °I–III with comorbidities (Table 3). These parameters were compared to the results of 636,490 out of 874,220 young male adults with under and normal weight (Table 2). To assess potential risk for CVD in this subgroup, the WHtR with a cut-off of 0.5 was used. Whereas the WHtR in young male adults with under or normal weight was elevated in 14.4% (*n* = 91,957; Table 2), the group of young male adults with overweight and obesity °I–III showed a significantly higher proportion of elevated WHtR with 75.1% (*n* = 178,583, *p* < 0.0001) and therefore higher risk for CVD. Furthermore, a significant and continuous increase of WHtR was observed in all BMI groups over the years (*p* < 0.0001) (Tables 2 and 3). Moreover, hypercholesterinemia (> 200 mg/dl) was significantly associated with overweight and obesity and elevated in about 16.2% (*n* = 38,527) of individuals with overweight and obesity (Table 3), compared to 5.5% (*n* = 35,203) of young male adults with under or normal weight (*p* < 0.0001) (Table 2). In the subgroup of young male adults with overweight and obesity, a significant association with hypertriglyceridemia (*n* = 64,781 of 237,730; 27.2%; *p* < 0.0001) and low HDL cholesterol concentration (*n* = 17,793 of 47,198; 37.7%; *p* < 0.0001) was found. An association of hypertension with elevated BMI > 25 kg/m^2^ was observed more frequently than in normal weight subjects (*n* = 37,120; 15.6%, *p* < 0.0001) (Table 3). Elevated systolic blood pressure was detected in 3.3% (*n* = 21,294) of young male adults with under or normal weight, compared to 15.6% (*n* = 37,953) of those with overweight or obesity (Tables 2 and 3).

**Table 2:** Descriptive evaluation of under and normal weight and associated comorbidities, *n* = 636,490 out of 874,220 (72.8%)

Year	Number *n* (%)	Elevated WHtR	Elevated cholesterol	Hypertension	Diabetes mellitus type 2		Smoking
2003	35,341 (78.7)	1,874 (5.3)	2,157 (6.1)	1,283 (3.6)	698 (2.0)	18,017 (51.0)	
2004	38,129 (76.7)	1,766 (4.6)	1,911 (5.0)	1,506 (3.9)	682 (1.8)	19,035 (49.9)	
2005	40,027 (75.3)	2,319 (5.8)	3,081 (7.7)	1,752 (4.4)	709 (1.8)	18,866 (47.1)	
2006	43,119 (74.6)	3,513 (8.1)	2,208 (5.1)	1,516 (3.5)	657 (1.5)	20,075 (46.6)	
2007	43,323 (73.9)	9,029 (20.8)	2,311 (5.3)	1,563 (3.6)	813 (1.9)	19,226 (44.4)	
2008	42,197 (73.1)	4,424 (10.5)	2,057 (4.9)	1,765 (4.2)	931 (2.2)	18,666 (44.2)	
2009	42,850 (73.0)	5,016 (11.7)	2,156 (5.0)	1,173 (2.7)	756 (1.8)	18,516 (43.2)	
2010	42,343 (72.0)	7,718 (18.2)	2,485 (5.9)	923 (2.2)	554 (1.3)	17,661 (41.7)	
2011	41,967 (72.1)	8,762 (20.2)	2,020 (4.8)	1,125 (2.7)	516 (1.2)	16,938 (40.4)	
2012	41,090 (71.3)	8,192 (19.9)	1,864 (4.5)	1,032 (2.5)	488 (1.2)	15,884 (38.7)	
2013	40,907 (71.9)	7,901 (19.3)	2,115 (5.2)	1,045 (2.6)	536 (1.3)	15,160 (37.1)	
2014	40,564 (71.5)	7,380 (18.2)	1,945 (4.8)	1,319 (3.3)	507 (1.2)	15,042 (37.1)	
2015	38,032 (71.4)	5,827 (15.3)	2,015 (5.3)	939 (2.5)	435 (1.2)	13,133 (34.5)	
2016	36,427 (71.1)	4,747 (13.0)	2,041 (5.6)	1,234 (3.4)	299 (0.8)	12,320 (33.8)	
2017	36,598 (70.1)	8,158 (22.3)	2,737 (7.5)	1,626 (4.5)	355 (1.0)	11,371 (31.1)	
2018	33,576 (69.0)	5,331 (15.9)	2,100 (6.4)	1,484 (4.4)	618 (1.8)	9,470 (28.2)	
Total	636,490	91,957 (14.4)	35,203 (5.5)	21,294 (3.3)	9554 (1.5)	259,380 (40.8)	

Abbreviations: *WHtR* waist-to-height-ratio

WHtR: > 0.5; elevated cholesterol: > 200 mg/dl; hypertension: ICD 9/10 and RR syst. > 140 mmHg; diabetes mellitus type 2: ICD 9/10 and elevated fasting blood sugar: > 125 mg/dl; smoking: yes

**Table 3:** Descriptive evaluation of overweight and obesity °I–III and associated comorbidities, *n* = 237,730 out of 874,220 (27.2%)

Year	Number *n* (%)	Elevated WHtR	Elevated cholesterol	Hypertension	Diabetes mellitus type 2		Smoking
2003	9,570 (21.3)	6,892 (72.0)	1,749 (18.3)	1,602 (16.7)	258 (2.7)	5,475 (57.2)	
2004	11,592 (23.3)	8,487 (73.2)	1,673 (14.4)	2,098 (18.1)	355 (3.1)	6,587 (56.8)	
2005	13,095 (24.7)	9,314 (71.1)	2,624 (20.0)	2,716 (20.7)	353 (2.7)	6,918 (52.8)	
2006	14,646 (25.4)	10,707 (73.1)	2,268 (15.5)	2,835 (19.4)	364 (2.5)	7,837 (53.5)	
2007	15,326 (26.1)	12,524 (81.7)	2,454 (16.0)	2,841 (18.5)	446 (2.9)	7,980 (52.1)	
2008	15,553 (26.9)	10,879 (69.9)	2,274 (14.6)	2,684 (17.3)	453 (2.9)	8,180 (52.6)	
2009	15,813 (27.0)	11,421 (72.2)	2,367 (15.0)	2,388 (15.1)	403 (2.5)	8,010 (50.7)	
2010	16,431 (28.0)	11,880 (72.3)	2,679 (16.3)	2,212 (13.5)	519 (3.1)	8,012 (48.8)	
2011	16,245 (27.9)	12,342 (76.0)	2,476 (15.2)	2,238 (13.8)	356 (2.2)	7,863 (48.4)	
2012	16,550 (28.7)	12,921 (78.1)	2,396 (14.5)	2,048 (12.4)	478 (2.9)	7,699 (46.5)	
2013	16,014 (28.1)	12,555 (78.4)	2,610 (16.3)	1,990 (12.4)	445 (2.8)	7,239 (45.2)	
2014	16,157 (28.5)	12,094 (74.9)	2,355 (14.6)	2,323 (14.4)	472 (2.9)	7,146 (44.2)	
2015	15,250 (28.6)	11,058 (72.5)	2,373 (15.6)	1,829 (12.0)	346 (2.3)	6,344 (41.6)	
2016	14,810 (28.9)	10,985 (74.2)	2,447 (16.5)	2,161 (14.6)	239 (1.6)	5,977 (40.4)	
2017	15,613 (29.9)	12,404 (79.4)	3,028 (19.4)	2,600 (16.7)	329 (2.1)	5,891 (37.7)	
2018	15,065 (31.0)	12,120 (80.5)	2,754 (18.3)	2,555 (17.0)	645 (4.2)	5,132 (34.1)	
Total	237,730	178,583 (75.1)	38,527 (16.2)	37,120 (15.6)	6,461 (2.7)	112,290 (47.2)	

Abbreviations: *WHtR* waist-to-height-ratio

WHtR: > 0.5; elevated cholesterol: > 200 mg/dl; hypertension: ICD 9/10 and RR syst. > 140 mmHg; diabetes mellitus type 2: ICD 9/10 and elevated fasting blood sugar: > 125 mg/dl; smoking: yes

In addition, a significant correlation of obesity and DM II was identified, ranging from 1.6% (*n* = 239) in 2016 to 4.2% (*n* = 645) in 2018 in young male adults with overweight and obesity (Table 3). Compared to 9,554 out of 636,490 (1.5%) young male adults with under or normal weight, fasting blood sugar was significantly elevated in those with overweight or obesity (*n* = 6,461 of 237,730; 2.7%; *p* < 0.0001) (Tables 2 and 3).

### Education and Socioeconomic Status

To evaluate the young male adults’ socioeconomic status, educational attainment and nicotine consumption were used. Interestingly, increased BMI correlated with higher numbers of school drop out (group 1a) or higher numbers of compulsory education only (group 1b) (Table 4). Overweight and obesity (°I–III) were significantly associated with low numbers of high school participation and graduation (*n* = 53,719 of 873,869; 6.1%) compared to young male adults with under and normal weight (*n* = 207,150 of 873,869; 23.7%; *p* < 0.0001). While 51,434 (31.5%) and 155,716 (32.8%) of young male adults with under and normal weight successfully graduated from high school, only 25.2% (*n* = 41,178) of young adults with overweight and 18.4% (*n* = 9,488), 15.3% (2,444), and 14.0% (*n* = 609) individuals with obesity °I, °II, °and III passed (group 2). Interestingly, 4.3% of young male adults with severe obesity (BMI > 40 kg/m^2^) dropped out of school before even finishing compulsory education (group 1a), 81.7% (*n* = 3,563) of those finished compulsory education only (group 1b). These results could be emphasized by comparing most deviating numbers of young male adults per BMI subcategory and educational status (*p* < 0.0001). Detailed data on the correlation of overweight and obesity and educational status are provided in Table 4.

**Table 4:** Educational status in dependence of the BMI distribution, *n* = 873,869 (%)

Status of education	Number *n* (%)	BMI < 20	BMI 20–25	BMI 25–30	BMI 30–35	BMI 35–40	BMI > 40	*p*-value*
1a*	21,132 (2.4)	4,195 (2.6)	10,381 (2.2)	4,140 (2.5)	1,641 (3.2)	586 (3.7)	189 (4.3)	*p* < 0.0001
1b*	591,868 (67.7)	107,698 (65.9)	309,267 (65.0)	118,052 (72.3)	40,353 (78.4)	12,935 (81.0)	3,563 (81.7)	*p* < 0.0001
2*	260,869 (29.9)	51,434 (31.5)	155,716 (32.8)	41,178 (25.2)	9,488 (18.4)	2,444 (15.3)	609 (14.0)	*p* < 0.0001
Total	873,869	163,327	475,364	163,370	51,482	15,965	4,361	

Abbreviations: *BMI* body mass index (kg/m^2^)

1a, no compulsory education; 1b, compulsory education but no high school graduation; 2, high school graduation or higher

^*^Comparing BMI and educational status of most deviating numbers of conscripts per category and row (*p* < 0.0001)

Nicotine abuse was detected significantly more often in 2018 in young male adults with overweight and obesity (*n* = 112,290; 47.2%, Table 3) than in normal weight subjects (*n* = 259,380; 40.8%, Table 2; *p* < 0.0001). Interestingly, the numbers of confirmed smokers with obesity decreased constantly between 2003 (*n* = 5,475; 57.2%) and 2018 (*n* = 5,132; 34.1%) (Table 3). Overall, 27.4% of young male adults with obesity (BMI > 30 kg/m^2^) were considered ineligible for military service due to their associated comorbidities.

## Discussion

By analyzing this nationwide data of almost all Austrian young male adults regarding weight, social background, and obesity-related comorbidities, we have shown for the very first time that the prevalence of 18-year-old males with overweight and obesity has been increasing over the past decades. Furthermore, increasing BMI is significantly associated with an elevated risk for CVD and poor socioeconomic and educational status. Especially the association between obesity and educational status is one major finding that highlights the impact of social behavior and education on nutritional status. These unique data regarding obesity and associated comorbidities are representative for trends in Western countries today.

### Weight and BMI

Obesity is a major burden; its prevalence has more than doubled within the past 30 years worldwide. The implications on children’s and adults physical and economic health are equally threatening [15]. As published by the Non-Communicable Diseases Risk Factor Collaboration (NCD-RisC), 38 million children under the age of 5 and more than 340 million youth between 5 and 19 years of age were estimated to be diagnosed with overweight or obesity in 2016 [16]. Gil et al. described increasing BMI trends throughout the Asia–Pacific region, affecting least developed as well as the wealthiest countries, such as Australia, Japan (+ 10% mean BMI; 16.7–24.0%) and China (+ 15.3%; 3.7–19.0%) equally [17, 18]. Furthermore, a continuous increase in the prevalence in childhood and adult obesity in low- and middle-income Asian countries was reported. However, data on participants’ height and weight was obtained simply by questionnaire [16].

Our data, acquired by standardized measurement techniques, show a significant increase in the prevalence of overweight and obesity in young adults over the past decades (Fig. 1). By comparing our data to a previously published study by Poglitsch et al., we were able to obtain similar results for an even longer time period of 16 years [13]. The results in this study are comparable with national surveys regarding weight and socioeconomic status and represent a trend in Western nations. Whereas a moderate increase of obesity °I and °II was noticed over the past years, a significant shift from normal to overweight was observed. As previously published, prevalence for overweight and obesity in Austria ranged from 15.5% and 5.8% in 2007 and increased to 29.0% and 8.4% in 2010. Data discrepancies among Poglitsch’s publication and our results can be explained due to further maintaining the database and statistical adjustments [13, 19]. In a nationwide survey performed in 2014, one quarter of young adolescents (15–29 years of age) were overweight, and 9.0% were obese [20]. Nevertheless, it has to be pointed out that exactly measured BMI, using a standardized protocol, was only reported by Poglitsch et al. and their study is therefore eligible for comparison to our results [13]. The present study showed a continuous increase of mean BMI and prevalence of overweight and obesity °I–III, rising from 15.3%, 4.2%, 1.2%, and 0.4% (2003) to 20.4%, 7.1%, 2.5%, and 0.8% (2018) (Table 1 and Fig. 2).

### Comorbidities

The association of obesity and an increased risk for a variety of associated comorbidities such as increased risk for CVD, hypertension, DM II, dyslipidemia, non-alcoholic fatty liver disease (NAFLD), developing malignancies, and autoimmune disease is well-known [15, 21–23]. Especially obesity in children and young adults is frequently accompanied by the risk of developing early-impaired glucose tolerance and insulin resistance, subsequently resulting in a risk to develop DM II or metabolic syndrome [24]. Most importantly, as described by Singh et al. [25], childhood obesity tracks further into adulthood, accompanied by an increased risk for CVD-associated mortality and morbidity, independent of adult BMI [26].

Obesity-related morbidity due to CVD accounts for about 70% of deaths in an observational period of 25 years [15]. The risk for cardio-metabolic comorbidity increases from double in overweight to more than ten times in people with severe obesity. As described by Canoy et al., abdominal obesity and smoking are both associated with an elevated risk for metabolic disease and CVD [27]. Further, a correlation of current smoking habits and increased tendency for abdominal obesity (e.g., elevated WHtR) was found [28].

Since data on WHtR as predictor for the risk of CVD and associated mortality is plausible, WHtR in association with BMI were applied as adequate parameters in the present analysis [5]. As indicated in the Bogalusa Heart Study, for instance, WHtR is strongly associated with adverse risk factor levels among children and adults and therefore an eligible indicator for risk assessment for future CVD in association with obesity and related comorbidities [29, 30]. The findings in this study show a significant association with increasing BMI and increasing CVD, using the WHtR as eligible tool (Tables 2 and 3). These results highlight the necessity for active screening and preventive measures to identify those at risk early [31]. Furthermore, significantly higher numbers of hypercholesterinemia, type 2 diabetes mellitus, and hypertension were diagnosed in young male adults with overweight and obesity (Tables 2 and 3). Continuously rising numbers of young male adults with overweight and obesity being affected by DM II over the past years have to be pointed out especially. Even though numbers of those identified with elevated fasting blood sugar or ICD 9/10 encoded DM II have been surprisingly low, a significant association of DM II and increasing BMI could be detected (Tables 2 and 3). Since DM II and obesity walk hand in hand with physical inactivity, adequate and early prevention tools, such as enhanced physical activity at school programs, should be of major interest. Furthermore, as published in a meta-analysis, focusing on the long-term follow-up of adolescents with severe obesity after metabolic and bariatric surgery, surgery turned out to be a safe and effective procedure in the treatment of morbid obesity and associated comorbidities such as diabetes mellitus [32]. Especially in adolescents with obesity suffering from type 2 diabetes mellitus, bariatric surgery reduced estimated CVD event risks significantly and was superior to medical therapy alone [33]. Unfortunately, it was not possible in our study to evaluate data on young male adults with obesity who have already received bariatric surgery at the time of compulsory military conscription examinations. Evaluating this data and demand for obesity surgery in future is of major interest for further evaluation.

### Interdependence of Socioeconomic Status and Obesity

Since obesity has increased substantially, especially in minority populations, socioeconomic and educational status of those affected by this “global epidemic” are obvious indicators [34]. In spite of this tremendous impact on socioeconomic parameters, exact numbers on prevalence are still largely missing. The WHO’s European office reported a strong relationship between obesity and lower socioeconomic and educational status, affecting women in particular [35].

In a multicenter study, including 103 low- and middle-income countries in an observational period of 21 years, Templin et al. described a substantial increase of overweight among the poorest population, whereas the prevalence was maintained by the wealthiest [36]. Talukdar et al. predict a continuous increase of global obesity prevalence especially in low- and middle-income countries within the next 5 years [37]. Next to the implementation of efficient national programs for disease prevention and promotion of health, profound therapy strategies for those being affected by the disease must be developed, implicating the broader acceptance of and easier access to the most powerful treatment of obesity and metabolic/bariatric surgery.

Besides the factor of wealth, quality of life and education are negatively associated with overweight and obesity as well. As described by Prause et al., health-related quality of life (HRQoL) [38], which is tightly connected with stress and physical and psychological well-being, is lower in this subgroup. The exact pathomechanisms responsible for this connection are not yet completely understood but might involve stress-related neuropeptide Y expression in the central amygdala that may cause insulin resistance, resulting in increased food intake and excessive development of obesity [39]. Furthermore, stress and other elusive behavior, such as nicotine consumption, were correlated positively, frequently resulting in food cravings and cravings for high fats, sweets, and carbohydrates.

Within the past years, all adolescents independent of their educational status were negatively affected by the decrease of physical and nutritional education. Since the beneficial effects of physical activity and a healthy diet on health and life expectancy are undeniable, enhanced measures in education, prevention, and elimination of these risk factors should be of major interest.

Due to the well-known benefit of physical activity regarding reduced levels of anxiety, stress, depression, and risk of non-communicable diseases, recommendations by the WHO and the European Union on physical activity per week have been announced in 2013 [40, 41]. Besides rapidly increasing prevalence of overweight and obesity, especially affecting children and young adults, high levels of insufficient physical activity (1/3rd) have been observed in the majority of European countries, particularly affecting those from low socioeconomic background, minority ethnic groups, and people with disabilities [42, 43]. On the basis of a national survey performed in 2014, 63% of young adults (18–29 years) fulfilled HEPA (health-enhancing physical activity) references [44] and 43% the WHO references in terms of sportive activities [20, 40].

Sanctions such as decreasing the overall intake of calories and sugared beverages were taken by the USA within the past decade. Furthermore, increasing the level of physical activity in high schools has brought an apparent plateau in the prevalence of obesity and diagnosed diabetes as described by Geiss et al. [45, 46]. For this purpose, similar measures taken by politics in other countries would be of interest.

Comparing these results to our data, similar results could be observed. As indicator for socioeconomic status, educational status and smoking habits were used as adequate parameters. Significantly less young male adults with overweight and obesity were attending and graduating from high school. Furthermore, a higher percentage of school dropouts was found in this observed subgroup. Also, smokers were found to be overweight or obese significantly more often (Table 4). Unfortunately, in our analysis, no data regarding conscripts’ fitness status were available. Nevertheless, increasing numbers of ineligibility for military service caused by obesity may be observed over the past 15 years and used as negative marker for physical fitness.

### Strengths and Limitations

We have addressed the major limitations of prior studies by including data of more than 95% of Austria’s male adolescents and quantifying the prevalence of overweight and obesity by using highly standardized measurements of height and weight. Since all young male adults are obliged to participate in the military’s clinical evaluation, the dropout rate could be reduced tremendously. To our knowledge, this represents the first exact evaluation of BMI and WHtR in young adults and a correlation with educational status and clinical history.

The demand for efficient public health improvements is undeniable, and educational measures regarding nutrition and physical activity should be prioritized. Furthermore, prevention tools should be implemented, assessing those at risk of developing obesity and associated comorbidities. Given these observations, an increased demand for effective treatment options for those affected by obesity, including multidisciplinary programs, pharmacotherapy, but metabolic/bariatric operations especially, could be expected over the next years. Potential limitations might be that only young, male Austrian adults were included in this study. Data was evaluated retrospectively, and since conscripts’ data was strictly anonymized prior to evaluation, further follow-up data on participant’s BMI and medical records, especially focusing on obesity-associated comorbidities as well as demand for bariatric surgery, were not available. Potential lack of data due to incomplete anamnesis could not be assessed subsequently due to prior anonymization of data. Furthermore, the use of mean BMI might not be ideal, since it might lead to a disproportionate increase in those at the extremes of the curve. Nevertheless, calculations for median BMI and t-testing showed similar results.

## Conclusion

Overall, the results of our study are alarming. We show that the prevalence of overweight and obesity in young male adults has been increasing over the past decades and is significantly associated with lower socioeconomic and educational status. BMI and risk for CVD have steadily increased over the last 15 years in Austrian male adolescents. A significant shift from normal to overweight was observed. Higher obesity classes showed an exponential increase. Consequently, efficient preventive measures and treatment strategies, such as structured multidisciplinary treatment programs, pharmacotherapy, but especially eased access to metabolic and bariatric surgery for those being affected, should be considered in the future.

## Data Availability

In terms of transparency, data and material will be provided if required.
